# The Main Determinants of Diabetes Mellitus Vascular Complications: Endothelial Dysfunction and Platelet Hyperaggregation

**DOI:** 10.3390/ijms19102968

**Published:** 2018-09-28

**Authors:** Albino Carrizzo, Carmine Izzo, Marco Oliveti, Antonia Alfano, Nicola Virtuoso, Mario Capunzo, Paola Di Pietro, Mariaconsiglia Calabrese, Eros De Simone, Sebastiano Sciarretta, Giacomo Frati, Serena Migliarino, Antonio Damato, Mariateresa Ambrosio, Francesco De Caro, Carmine Vecchione

**Affiliations:** 1 IRCCS Neuromed, 86077 Pozzilli, IS, Italy; albino.carrizzo@gmail.com (A.C.); sebastianosciarretta@yahoo.it (S.S.); fraticello@inwind.it (G.F.); antonio.damato85@libero.it (A.D.); mattyambr@gmail.com (M.A.); 2 Departement of Medicine and Surgery, University of Salerno, 84081 Baronissi, SA, Italy; carmine.izzo93@gmail.com (C.I.); olivetimarco@yahoo.it (M.O.); capunzom@virgilio.it (M.C.); p.dipietro06@virgilio.it (P.D.P.); fdecaro@unisa.it (F.D.C.); 3 Heart Department, A.O.U. “San Giovanni di Dio e Ruggi d’Aragona”, 84131 Salerno, Italy; antonia.alfano@sangiovannieruggi.it (A.A.); eros.desimone@sangiovannieruggi.it (E.D.S.); 4 Department of Cardiovascular Medicine, A.O.U. Federico II, 80131 Naples, Italy; n.virtuoso@hotmail.com; 5 Rehabilitation Department, A.O.U.“San Giovanni di Dio e Ruggi d’Aragona”, 84131 Salerno, Italy; mac.calabrese@virgilio.it; 6 Department of Medico-Surgical Sciences and Biotechnologies, Sapienza University of Rome, 00161 Rome, Italy; 7 Department of Clinical and Molecular Medicine, School of Medicine and Psychology, Sapienza University of Rome, 00161 Rome, Italy; smigliarino2018@virgilio.it

**Keywords:** diabetes, endothelial dysfunction, platelets activation, molecular mechanisms

## Abstract

Diabetes mellitus is a common disease that affects 3–5% of the general population in Italy. In some countries of northern Europe or in North America, it can even affect 6–8% of the population. Of great concern is that the number of cases of diabetes is constantly increasing, probably due to the increase in obesity and the sedentary nature of the population. According to the World Health Organization, in the year 2030 there will be 360 million people with diabetes, compared to 170 million in 2000. This has important repercussions on the lives of patients and their families, and on health systems that offer assistance to patients. In this review, we try to describe in an organized way the pathophysiological continuity between diabetes mellitus, endothelial dysfunction, and platelet hyperaggregation, highlighting the main molecular mechanisms involved and the interconnections.

## 1. Introduction

Diabetes mellitus is a multiple-etiology metabolic disorder characterized by chronic hyperglycemia, with alterations in carbohydrate, fat, and protein metabolism due to defects in secretion and/or insulin action [1]. This disease is defined as a heterogeneous syndrome, because it includes various clinical forms, of which the most important are type 1 or insulin-dependent diabetes mellitus, caused by an autoimmune destruction of β cells, and type 2 diabetes mellitus or insulin-independent, characterized by a partial deficiency of insulin secretion and often established on the condition of insulin resistance [2].

The main factors causing diabetes (DM) are: the increase in cases of obesity, the increase in the average age and life expectancy, a more sedentary lifestyle, the increase in stress, and above all, genetics. Actually, DM is considered to be a public health problem of epidemic proportions, recognized as an independent risk factor for cardiovascular disease (CVD), even when under glycemic control [3,4].

Although diabetes mellitus is still an incurable disease, some therapies allow those affected to lead a lifestyle that is as normal as possible. Since the discovery of insulin in 1921, the prognosis of diabetic disease has changed, turning it from acute and fatal to chronic; the average life of a type 1 diabetic was once a few months, with death following ketoacidosis or infections. In the early years of its discovery, insulin therapy, although capable of saving lives for people with diabetes, was not able to protect them from the development of diabetic complications. In fact, since diabetes is associated with innumerable collateral alterations, it still represents one of the primary causes of death worldwide [4]. Type 2 diabetes (T2DM) is the most widespread endocrine-metabolic disorder in the world, affecting 5–10% of the population of industrialized countries and accounting for about 90% of cases of diabetes mellitus. According to WHO assessments, the annual growth of diabetes is estimated at 5–6%.

The association between diabetes and cardiovascular and cerebrovascular diseases is well known: angina pectoris, myocardial infarction, stroke, peripheral arteriopathy, arterial thrombosis, and venous thrombosis represent the most frequent forms of circulatory alterations, mainly associated with atherosclerotic processes and alterations of the blood flow. It is estimated that around 5.2% of cardiovascular deaths are associated with diabetes [5]. This brief epidemiological premise is fundamental to introduce the concept of diabetes as a risk factor for thrombotic disease and vascular endothelial alterations.

Actually, injuries induced by DM on micro- and macrovessels are considered the main causes of increased morbidity and mortality in this disease and, recently, novel experimental treatments have been proposed to reduce the negative effect of DM on vascular function [3,6,7]. Since it is widely accepted that in DM there is an impairment of endothelial nitric oxide synthase (eNOS) activity as well as enhancement of production of reactive oxygen species (ROS), resulting in diminished nitric oxide (NO) bioavailability and the consequent vascular alterations [4,8], several experimental studies have focused on possible pharmacological treatment aimed to restore this alteration alone [3,9,10]. However, increasing investigations have demonstrated that the basal proinflammatory state in diabetes seems to play a fundamental role in the interconnection between endothelial dysfunction and platelet hyperaggregability. In fact, during DM, there is the exposure of several endothelial dysfunctional markers, such as vascular cell adhesion molecule (VCAM)-1, von Willebrand factor (vWF), C-reactive protein (CRP), and tumor necrosis factor (TNF)-alpha [11], that are able to drive platelet hyperaggregation. Moreover, it has been demonstrated that patients with diabetes mellitus have significant alterations in plasma protein coagulation levels so that the disease can be considered itself a condition of hypercoagulability [12,13]. In fact, it has been shown that the levels of VII, FVIII, and vWF factors and fibrinogen are significantly higher in diabetic patients than in the general population, thus suggesting an important role of hypercoagulability in the disease [14]. Although there are different mechanisms that induce the process of endothelial dysfunction and platelet aggregation in diabetes, hyperglycemia is the main mediator of these events. In fact, starting from the endothelial damage induced by the alteration of the biochemical and hemodynamic mechanisms, tissue factor (TF) is released, which guides the platelets towards the hyperaggregation process. Until now, several studies have focused mainly on endothelial dysfunction or on platelet hyperaggregability in diabetes. In this review, we have tried to collect all literature available to date on these diabetic determinants, highlighting the main interconnections. 

## 2. Endothelial Dysfunction and Diabetes

Although only a thin monocellular layer, healthy endothelium is optimally able to respond to chemical and physical signals by the production of a wide range of factors that regulate cellular adhesion, smooth muscle cell proliferation, vascular tone, thromboresistance, and vessel inflammation. Vascular endothelium is an active endocrine, paracrine, and autocrine organ. It is indispensable for the regulation and maintenance of vascular tone and homeostasis [15]. Endothelial dysfunction is an alteration of normal endothelial function, which implies the loss of some structural and/or functional features and represents one of the most important determinants of CVD. Endothelial dysfunction is characterized by a reduction in the bioavailability of vasodilators, particularly nitric oxide (NO) [16], and/or an increase in endothelium-derived contracting factors, like angiotensin II (Ang II). This vasomotion plays a direct role in the balance of tissue oxygen supply and the metabolic requirements regulating vessel tone and diameter, and it involves the remodeling of vascular structure and long-term organ perfusion [17]. It is widely accepted that endothelial dysfunction is a mechanism that potentially unifies the etiology of diabetes and CVD, and it could contribute to insulin resistance, leading to diabetes [17]. Several markers and processes have revealed the alteration of endothelial function, such as elevated plasma levels of von Willebrand factor (vWF), plasminogen activator inhibitor-1 (PAI-1), or cellular adhesion molecules [18,19,20]; impaired flow-mediated vasodilatation of the brachial artery [21]; altered forearm blood flow or vasodilatation in the forearm skin [22]; and the presence of retinal arteriolar narrowing [23]. Since all cross-sectional studies of patients with T2DM [24,25] have detected an endothelial alteration, the establishment of endothelial dysfunction as a fundamental precursor to type 2 diabetes may reveal new avenues for diabetes prevention and treatment. 

### Pathophysiology and Molecular Signaling in Diabetic Endothelial Dysfunction

Under normal homeostatic conditions, with no expression of proinflammatory factors, the endothelium maintains normal vascular tone and blood fluidity. However, cardiovascular risk factors, including smoking, aging, hypercholesterolemia, hypertension, and hyperglycemia, are all associated with an alteration of normal endothelial function [15,26]. This mechanism leads to a chronic inflammatory process, a loss of antithrombotic factors, and an increase in vasoconstrictor and prothrombotic products, promoting an abnormal vasoreactivity, therefore elevating the risk of cardiovascular events [27]. Vascular endothelium is a major target of oxidative stress, playing a critical role in the pathophysiology of several vascular diseases and disorders, and its alteration significantly contributes to diabetic vascular pathology. It is well known that hyperglycemia is able to cause endothelial dysfunction [28,29]: most observations suggest that the damage from hyperglycemia on endothelium is secondary to oxidative stress, but the data available in the literature seem to highlight more mechanisms that are heterogeneous and too complex to explain endothelial damage from hyperglycemia. It is important to underline that endothelial dysfunction is related to specific physiopathological mechanisms that involve: alterations of the substrate/enzyme ratio; alterations in the expression/structure of NOS; signal alterations; and alterations of the availability of cofactors and destruction of NO [30,31]. The first hypothesis is supported by both clinical and experimental evidence that has amply demonstrated how, by increasing the substratum for NOS, l-arginine, there is an improvement of endothelial function in terms of NO production [32,33]. Alternatively, compounds that inactivate or alter the normal precursor/product ratio have been highlighted: one of these is asymmetric dimethyl arginine (ADMA). It has recently been found that, in type 2 diabetes, ADMA increases significantly after ingestion of a fatty meal, and its levels correlate with the reduction of flow-mediated vasodilatation [34]. Regarding the second hypothesis, it has been shown that some factors are able to reduce the expression of NOS. Among these are the hyperglycemia itself, hypoxia, high concentrations of TNF alpha, and high concentrations of low-density lipoprotein (LDL) oxidized. All these conditions characterize the metabolic milieu of a type 2 diabetic [35]. In vitro studies have reported that, in the diabetic condition, there are alterations of the NO signal that compromise the physiological endothelium vasodilatation, thus contributing to the alteration of macro- and microcirculation. Moreover, NO, in addition to being a vasodilator, also reduces vascular permeability and the monocyte and lymphocyte adhesion molecules’ synthesis, contributing to the reduction of tissue oxidation, tissue inflammation, platelet aggregation, and activation of thrombogenic factors, leading to a reduction of typical inflammatory processes induced by hyperglycemia. For all these reasons, NO is considered an important antiatherogenic molecule [15,36,37] that is necessary to contain the diabetic endothelial alterations. Unfortunately, the eNOS enzyme, which normally helps maintain the quiescent state of the endothelium, can switch to generating ROS in appropriate circumstances as part of endothelial activation. It has been reported that this enzyme is the key center of endothelial homeostasis, as it can regulate both the quiescent and activated endothelial status. In determinate occasions, chronic production of ROS may exceed the capacity of cellular enzymatic and nonenzymatic antioxidant molecules and thus contribute to diabetic vascular disease through the induction of sustained endothelial activation. Cellular injury and increases in the permeability of endothelium, due to reactive oxygen species (ROS) at higher concentrations, represent one of the main causes of vascular alterations in diabetes [38,39]. Important sources of oxidative stress in the endothelium, particularly during diabetes, are NADPH oxidases and xanthine oxidase (XO), which have been shown to have increased activity in arteries from patients with DM and related CVD [40,41]. Recent studies have demonstrated that Rac1—a small GTPase protein—is actively involved in diabetic vascular alteration. In particular, both the genetic silencing that the pharmacological inhibition of Rac1 results in a significant improvement of NO signaling and in the reduction of NADPH activity, restoring the physiological vasorelaxation without modifying the blood glucose levels in animal models [3,10]. 

Several studies have demonstrated that the interaction between ROS and NO sets up a vicious circle, which results in further endothelial injury, activation, and inflammation. These, protracted in time, can make endothelial cells (ECs) lose integrity, go into senescence, and detach from the vessel into the circulation. Activated or apoptotic ECs, if damaged, release endothelial microparticles that we can view as circulating markers [42]. It has been demonstrated that circulating total microparticles are greatly elevated in patients with T2DM, suggesting that changing microparticle levels are possibly relevant to diabetic conditions [43], thus opening a new scenario for future investigations. Recently, it has also been demonstrated that Sterol regulatory element-binding protein 1 (SREBP1)—an important transcriptional regulator of lipogenesis [44], regulated by Sec23A [45]—is involved in the modulation of insulin resistance and in the lipogenesis that is chronically enhanced diabetes, thus laying the foundation for the development of novel future therapeutic strategies to contain the onset of diabetes.

Clearly, endothelial integrity not only depends on the injury extent but also on the endogenous capacity of repair. In this regard, circulating endothelial progenitor cells (EPCs) play an important role in diabetes, since they are able to activate the reparative processes of endothelium [46]. Generally, EPCs, recruited from the bone marrow, reach peripheral blood circulation, in which they can differentiate into mature cells with endothelial characteristics. In the diabetic condition, the clinical severity of occlusive vascular disease in diabetic subjects has been partly attributed to an altered development of collateral vessels. EPCs from diabetic patients have been shown to exhibit altered proliferation, adhesion, and incorporation into vascular structures [47]. Therefore, a reduction in EPCs could be a mechanism through which individuals with diabetes have a reduced ability to form collateral vessels. Mechanisms involved in the qualitative and quantitative alterations of EPCs in diabetes mellitus are currently not fully known, and data in the literature are few. What is known so far is that hyperglycemia is known to induce oxidative stress and ROS production in vivo, which are able to inhibit the proliferation and the function of EPCs concomitant with the reduction of NO synthesis and matrix metalloproteinase 9 (MMP-9), which are necessary for the mobilization of these cells by the bone marrow [48]. Based on these results, the correlation between NO, ROS, reparative processes, and EPCs require further investigation in relation to diabetes.

**Figure 1:**
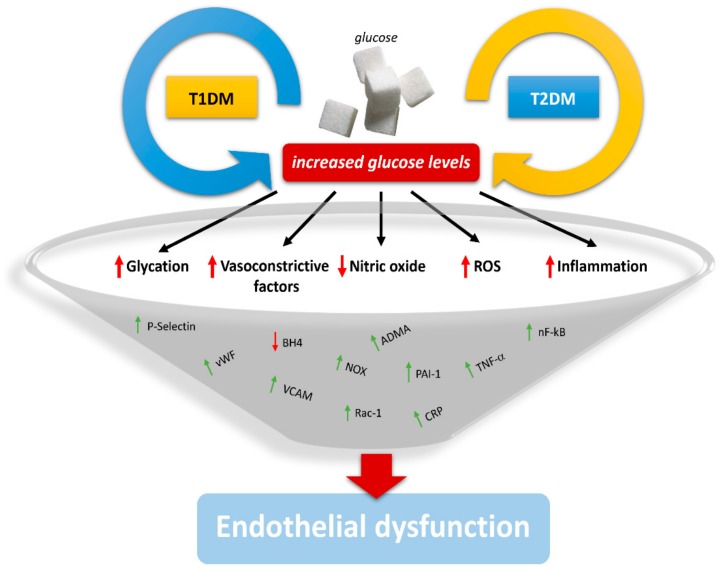
Schematic representation of the main processes and molecules involved in the pathogenesis of endothelial dysfunction in diabetes. The sustained hyperglycemia during diabetes increases glycation through advanced glycation end products (AGEs); promotes the production of vasoconstrictive factors, such as asymmetric dimethyl arginine (ADMA); reduces the bioavailability of nitric oxide (NO) and BH4, increasing reactive oxygen species (ROS) formation through Rac1 and NOX recruitment and increasing the inflammatory status through the expression of several inflammatory molecules such as tumor necrosis factor (TNF)-α, P-selectin, vascular cell adhesion molecule (VCAM), plasminogen activator inhibitor-1 (PAI-1), von Willebrand factor (vWF), C-reactive protein (CRP), and nF-Kβ.

As reported above, DM has many ways of leading to endothelial dysfunction. The increased oxidative stress, the alteration of lipogenesis, the reduction of nitric oxide, and the alteration of EPC function create what is called the diabetic state (Figure 1), in which the alterations of the vessel wall lead to the pathogenesis of arterial thrombus. The damage of the vessel wall (for example, the formation of atherosclerotic plaque) involves a cascade of events that progressively determine the thickening of the vascular wall, with consequent modification of the blood flow increasing platelet aggregation [49,50].

## 3. Platelet Aggregation in Diabetes Mellitus

Diabetes mellitus is associated with an increased risk of vascular disease. Patients with diabetes mellitus type 2 show an increased reactivity to and baseline activation of platelets, thus increasing events such as thrombosis due to atherosclerotic plaque rupture. Atherosclerotic plaque rupture exposes subendothelial material, inducing platelet activation initiating the coagulation cascade and the formation of thrombus. Platelets in patients with diabetes mellitus show dysregulated signaling pathways with platelet hyperactivation, with a consequent increase in microcapillary embolization and accelerated local vascular lesions [51,52].

### 3.1. Biochemical Factors in Diabetic Platelet Dysfunction

Postprandial hyperglycemia is the main diagnostic element that associates DM to macro- and microvascular disease and thus to increased cardiovascular risk and an increased prothrombotic state [53,54]. Studies show an increase in soluble P-selectin and CD40-ligand in acute hyperglycemia in healthy subjects, who show increased platelet reactivity and platelet activation [55,56,57]. The same effect can be elicited by exposing platelets to hyperosmolar solutions, miming hyperglycemia’s direct osmotic effect [58]. In vivo increased protein kinase C (PKC) levels, an indicator of the proaggregatory state, have been shown both in chronic and acute hyperglycemia [59]. On the other hand, unlike healthy individuals’ platelets, patients with DM have an inherited-like diabetes-related pathway dysregulation, highlighted by the short-term activation of calcium-sensitive PKCβ isoenzyme at the basal vitro state, as if in presence of an acute hyperglycemic state. From another perspective, improvements in glycemic control have been associated with reduced platelet reactivity in patients with type 2 DM, thus indicating a cause and effect relationship [60]. In fact, in type 2 DM patients, the maintenance of optimal percutaneous coronary intervention (PCI) preprocedural glycemic control (glycated hemoglobin (HbA1c) < 7%) is associated with improved clinical outcome, while mild elevation of preprocedural fasting glucose is associated with increased risk of mortality [61,62].

Hyperglycemia-induced nonenzymatic interactions between the carbonyl groups of the reducing sugar and the primary amino group lead to the production of advanced glycation end products (AGEs) [63]. AGEs include a heterogeneous group of compounds, some of which cause clotting factor activation by externalization of platelet membrane phosphatidylserine, thus inducing a direct thrombogenic state [64]. By the same process, increased glycation levels of surface membrane proteins of the platelets in patients with DM decrease membrane fluidity and enhance platelet sensitivity to agonists [65,66].

The platelet activation signaling pathway is mediated ultimately by glycoprotein IIb/IIIa receptor (GPIIb/IIIa) platelet–fibrin interaction, which mediates binding to von Willebrand factor. Expression of platelet surface GP IIb/IIIa, as well as that of GPIb, is correlated to HbA1c levels. In fact, hyperglycemia leads to release of larger platelets with more GPIb and GPIIb/IIIa receptors, thus increasing the aggregation baseline activation and the thromboxane forming capacity [67].

P2Y12—another platelet surface receptor and a target of the thienopyridine antiplatelet agents—has also been shown to be increased on platelets in patients with DM due to membrane fluidity dynamics alteration [68]. P2Y12 receptor activation leads to suppressed cyclic adenosine monophosphate (cAMP)-dependent phosphorylation of vasodilator-stimulated phosphoprotein (VASP-P) mediated by specific protein kinases (PKA), ultimately leading to increased activation and aggregation of platelets [69]. Patients with DM have been shown to have lower cAMP platelet levels and a higher P2Y12 signaling level compared to nondiabetics. Moreover, compared to nondiabetic patients, higher baseline intracellular calcium levels have been found in platelets of older diabetics, especially in response to thrombin agonism [68,70]. Finally, platelets with lower cAMP levels and higher baseline calcium are more susceptible to activation and aggregation at lower levels of agonist stimulation.

Lipid metabolism can also alter platelet function. Hypertriglyceridemia and low levels of high-density lipoprotein (HDL) can commonly be found in patients with impaired glucose homeostasis. Moreover, hypertriglyceridemia can lead to high levels of very low-density lipoprotein (VLDL). This lipidic profile potentiates platelet activity by apolipoprotein E and its interaction with the platelet LDL receptor [71]. In patients with DM, administration of reconstituted HDL has been shown to suppresses aggregation by promoting cholesterol efflux from platelet membranes [72]. A further contribution to platelet hyperreactivity, however, is mediated by lipids and glucose interaction with low-density lipoprotein (LDL) formation, which leads to impaired nitric oxide production and increased intraplatelet calcium concentration [73].

### 3.2. Insulin Effects on Platelets in Diabetes

As discussed previously, type 2 DM represents about 90% of all DM cases and is mainly referred to as a pathology caused by reduced tissue sensitivity to insulin. Insulin resistance is initially compensated by increased pancreatic β-cells insulin production in order to maintain fasting euglycemia; this is referred to as a prediabetic stage. Pancreatic β-cells, in susceptible individuals, as a result of the chronically increased insulin demand, initially undergo hypertrophy and subsequent apoptosis, leading to a reduction in absolute β-cell mass. In the long run, early stages of DM2 with hyperinsulinemia progressively become relative and eventually absolute insulin deficiency.

Human platelets have an insulin receptor (IR) which can directly regulate platelet function [74]. These effects, however, are still to be well defined. In particular, in healthy nonobese patients, the binding of insulin to its platelet receptor leads to magnesium intracellular translocation, thus decreasing thrombin-induced platelet aggregation and reducing production of proaggregatory thromboxane B2 [75]. This effect is mediated by activation of insulin receptor substrate 1 (IRS-1) by tyrosine phosphorylation and a relative Giα-subunit reduced activation with consequent increased cAMP intraplatelet levels, reduction of P2Y12 signaling, and reduced platelet activity [76,77]. The IR acts in association with the insulin-like growth factor 1 (IGF-1) receptor, defining the magnitude of such effects [78]. The downstream mediator of the IR, insulin receptor substrate 1 (IRS-1) and IRS-2, also act in association with the IGF-1 receptor, abundantly expressed in platelets, increasing platelet reactivity [79]. These studies confirm that in healthy nonobese individuals, insulin inhibits platelet interaction with collagen, decreasing platelet aggregation [80,81].

On the basis of these pieces of evidence, it may seem logical to presume that DM type 1 patients, who have absolute insulin deficiency, should have increased platelet reactivity, while DM type 2 patients, who have hyperinsulinemia, should have suppressed platelet activity. Well, this is far from the truth, as DM type 2 patients are commonly obese, and this induces insulin resistance, which we know is associated with platelet hyperreactivity [82]. In fact, in obese insulin-resistant patients without DM, a euglycemic hyperinsulinemic clamp fails to suppress platelet activity [82]. More interestingly, increased plasma CD40L, increased levels of platelet-derived microparticles (released in blood by platelet activation), and higher thromboxane production can be found in obese patients [83,84,85]. Finally, in obese women, a study showed how on the other hand insulin sensitization, by pioglitazone administration or weight loss, reduces markers of platelet activation [83,86].

The insulin receptor signaling pathway alteration in patients with insulin resistance versus healthy individuals is as evident in tissues as it is in platelets [68]. Reduction in platelet insulin sensitivity, as described before, leads to platelet hyperreactivity due to a reduction in cAMP levels and a consequent increased intraplatelet calcium concentration [68,87]. These effects can be also seen in patients with type 2 DM in insulin therapy with a paradoxical increase in platelet reactivity [88]. Of interest is the IRS-independent impairment of sensitivity to prostacyclin and nitric oxide in platelets of DM patients, leading to an increased platelet reactivity [89,90]. Therefore, hyperinsulinemia is a harmful factor for platelet reactivity in patients with insulin resistance. Not only does insulin increase platelet reactivity, but in association with hyperglycemia it also increases levels of tissue factor procoagulant activity, decreases levels of factor VII/VIIa, and increases factor VIII and prothrombin fragment F1.2, ultimately bringing about a procoagulant state [57]. A distinctive sign of insulin platelet activation pathway is the upregulated platelet expression of CD40L and increased monocyte–platelet aggregates, which differs from other agonists [91].

### 3.3. Effects of Oxidative Stress and Inflammation on Platelet Function

Patients with DM have a higher baseline of oxidative stress and inflammation levels compared to healthy subjects. The overproduction of reactive oxygen and nitrogen species and potent radicals in patients with DM is the direct cause of increased platelet activation [92,93,94]. Interestingly, at the basal state and even greater in acute hyperglycemic episodes, DM patients show an increased level of 8-iso-prostaglandin F2α (8-isoprostane), a marker of an oxidative stress product of nonenzymatic arachidonic acid peroxidation [95,96,97]. The mechanism behind increased platelet aggregation and reactivity associated with oxidative stress is the direct intraplatelet calcium release in response to superoxide anion production [98].

**Figure 2:**
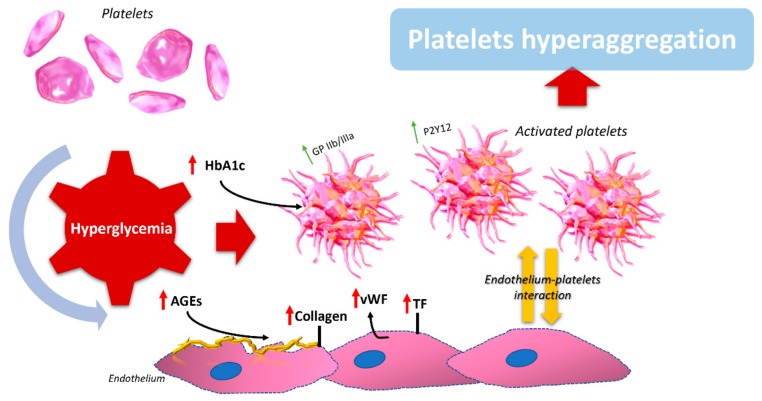
Schematic of the main processes involved in platelet hyperaggregation in diabetes. The hyperglycemic condition regulates several molecular mechanisms both at endothelial and platelet levels. The high circulating levels of AGEs and the vessel wall damage lead to the increase of collagen exposition, von Willebrand factor (vWF), and tissue factor (TF). Concomitantly, the increased level of glycated hemoglobin (HbA1c) induces the expression on platelet surface of different glycoprotein receptors (GPIIb/IIIa), promoting platelet hyperactivation.

An increased rate of advanced glycation end product (AGE) production is highlighted during recurrent episodes of hyperglycemia, as reactive oxygen species enhance the interaction of sugars with proteins. Moreover, the interaction of reactive oxygen species with AGE receptors (RAGEs) on the endothelium is a cause of endothelial dysfunction and thus leads to an increased inflammatory state [99]. NF-κB is the main key RAGE mediator, and by its translocation into the nucleus it determines the increase in prothrombotic and proinflammatory genes, which starts a vicious cycle [100]. This is true for the endothelium, but not for platelets, as they are missing a nucleus. So, although RAGEs do influence platelet activity, it is done indirectly. Recent studies have highlighted as a possible direct mechanism linking AGEs and platelet hyperreactivity the increased expression of CD36, CD62, and CD63 on the platelet surface membrane [101,102]. These receptors have been associated with enhanced platelet reactivity in vitro, as well as enhanced arterial thrombosis in vivo. In the blood stream of diabetic patients, another stimulus for clot formation is AGE-mediated enhanced macrophage-1 antigen (Mac-1) expression on neutrophils. In particular, expression of Mac-1 on neutrophils has been shown to enhance platelet–neutrophil aggregates and induce expression of TF by monocytes, thus highlighting the importance of AGEs in thrombosis and in the inflammatory process in diabetic patients [103]. Another interesting piece of evidence is that plasma serotonin increases in diabetic patients. This increase is mainly due to platelet hyperactivation by AGEs, which have been shown to have a dose-dependent relation [104]. All the previous findings have been also demonstrated on a sample of 50 patients (22 with type 1 diabetes, 21 with type 2 diabetes, and 7 healthy subjects), thus confirming the relation between AGEs and platelets hyperactivation [101]. Interestingly, in a study on 20 type 2 diabetic patients after a high-AGE content meal administration showed an increase in endothelial dysfunction (in terms of increased E-selectin, increased vascular cell adhesion molecule-1, and increased intracellular adhesion molecule-1 levels) and oxidative stress. This study demonstrates how a high-AGE meal is able to induce an acute impairment of vascular function compared to a low-AGE meal, highlighting how chemical modification of cooking food plays an important role in micro- and macrovascular function [105]. In short, oxidative stress induced by AGEs causes a reduction in nitric oxide (NO) production, which normally inhibits platelet activation, thus leading to platelet hyperreactivity [106,107]. Finally, a study performed on diabetic patients with different stages of chronic disease compared with healthy subjects clearly demonstrates the direct link between endothelial alterations, inflammation, and AGEs, showing that high circulating levels of advanced glycation end products mediate endothelial dysfunction [108]. All these effects, combined with the accelerated turnover of platelets in patients with DM, increased platelet size, increased hyperreactiveness, and reduced effect of antiplatelet therapy significantly promote the progression of vascular diabetic complications (Figure 2) [109,110].

### 3.4. Clinical Implications of Platelet Hyperactivity in Diabetes

As of today, the antiplatelet therapy based on low-dose aspirin remains the only way to reduce, by 20%, myocardial infarction (MI), stroke, or cardiovascular death risk in patients at intermediate-to-high risk [111]. However, in vitro laboratory tests have highlighted that, despite aspirin therapy, platelets from diabetic patients react to various platelet agonists, leading to an increased atherothrombotic risk [112,113,114,115,116]. Moreover, biochemical testing on residual platelet reactivity in DM patients on aspirin therapy showed a residual activity of 10–40%, which is alarming evidence [117,118]. This reduction in the effectiveness of aspirin achieving its expected pharmacological effect—inhibiting the conversion of arachidonic acid to thromboxane A2 (TXA2) by the platelet cyclooxygenase-1 (COX-1) enzyme—is variable and only amounts to about 5% [119,120].

More than an “Aspirin Resistance”, in fact, it should be defined as a “high on-treatment platelet reactivity” (HTPR), where platelet activation persists due to agonist-induced activity, despite aspirin therapy. The local positive-feedback loop that amplifies the platelet activation is due to activated platelet eicosanoid thromboxane A2 (TXA2), which sensitizes platelets to agonists and activates bystander quiescent platelets. By inhibition of COX-1, aspirin interferes with the thromboxane feedback loop, limiting platelet response to weak agonists (ADP and collagen) and leaving partially active potent agonists (thrombin) which can still induce platelet activation [121].

Overall, patients with DM who have hyperreactive platelets, as discussed previously, also manifest HTPR, ultimately greatly increasing the risk of future thrombotic events. Taking the previous into account in patients at high risk of thrombotic events, such as patients with acute coronary syndrome (ACS) or undergoing PCI, aspirin monotherapy may not be enough, and we should in these cases consider a dual antiplatelet therapy [122]. However, such an evaluation in patients with chronic, stable DM, as well as in patients with stable coronary, cerebrovascular, or peripheral vascular disease, did not show a reduction of MI, stroke, or cardiovascular death events after administration of dual antiplatelet therapy (DAT) (aspirin plus clopidogrel vs. aspirin alone, CHARISMA trial) [123]. Furthermore, DAT has been associated with increased hemorrhagic events, reducing the therapeutic index [124,125].

In conclusion, primary prevention in DM patients with aspirin has, as of today, no real evidence. However, statements of the American Diabetes Association suggest an aspirin primary prevention in DM patients who are at a >10% 10-year risk of cardiovascular disease or in patients at intermediate (5–10%) risk [126].

## 4. Interactions between Endothelial Dysfunction and Platelet Hyperaggregation in Diabetes

As described so far, besides comorbidities, such as atherosclerosis and arterial hypertension, in diabetic patients, there two early changes that contribute, with multiplicative effect, to the development and progression of vascular complications, such as endothelial dysfunction and platelet hyperaggregation.

In order to prevent platelet aggregation and adherence to the endothelium, healthy vessels continuously produce NO and PGI2 [127]. These antiaggregants are regulated by a negative feedback mechanism set by the level of platelet aggregation in response to many different factors, such as platelet-released serotonin, plasma thrombin, platelet-derived growth factor, interleukin-1, bradykinin, and ADP in order to limit the platelet plug growth in the area of damage of the vessel [128]. In detail, prostacyclin binds to a seven-transmembrane domain structure receptor linked to a G-protein inhibitory (Gi) for adenylate cyclase, which in turn is linked to the α2-adrenergic receptor, which ultimately binds to epinephrine [129,130]. On the other hand, NO, due to its short life span and small size, directly activates guanylate cyclase by diffusing across the platelet membrane. In both cases, the end result is an inactivation of the platelet proteins crucial for aggregation mediated by phosphorylation of cAMP-dependent and cGMP-dependent protein kinases [127,131]. These molecules are of great interest, as many animal studies have shown decreased vascular synthesis and release of both NO and PGI2 in diabetic rat models [132,133,134,135,136]. Moreover, metabolic control with insulin or pancreatic islet transplantation in the same diabetic rat models have shown a return to normal of vascular PGI2 and NO synthesis [131]. Therefore, considering the data previously reported, it seems evident that increased platelet activity derives from impaired synthesis and release of antiaggregants from endothelium, as well as from reduced platelet sensitivity to antiaggregants in diabetes. In fact, in diabetic patients with vascular diseases, platelets have been shown to respond less to PGI2 and NO [137,138]. This diminished platelet sensitivity to NO and PGI2 can be due to various mechanisms, such as impaired receptor activity and/or reduction of receptor number. In this regard, it has been demonstrated that PGI2 receptor activation by prostaglandin E1 determines a reduced adenylate cyclase stimulation. Interestingly, a human trial on insulin administration to 10 non-diabetic lean subjects and 10 obese insulin-resistant subjects has shown that only in the platelets of lean non-diabetic subjects was there an increased cAMP response to PGI2, thus corroborating the alteration of the PGI2 receptor [49,139,140]. Unfortunately, further studies are needed to understand the mechanisms that underlie the impaired cAMP response.

Another link between endothelial dysfunction and platelet hyperaggregation in diabetes mellitus is the increased release of von Willebrand factor, a glycoprotein released into the circulation by secretion from endothelial cells. It has been demonstrated that damage endothelium releases high levels of vWF [141]. Interestingly, several studies have reported that diabetic patients show high plasma levels of von Willebrand factor [142]. This glycoprotein is able to bind GPIb-IX and IIb-IIIa platelet receptors, promoting platelet aggregation.

Moreover, a recent study demonstrates that Rac1, a small GTPase, is involved both in endothelial and in platelet dysfunctions in diabetes. In fact, the inhibition of Rac1 in animal and human models showed a rescue of altered endothelial function and a reduction of platelet hyperaggregation. Interestingly, this inhibition was able to exert an additional effect on ASA-treated platelets, clearly demonstrating its key role in linking endothelial dysfunction and platelet hyperaggregation in diabetic pathology [3].

Last but not least, the process that clearly links endothelial dysfunction with platelet hyperactivation is represented by AGEs. In fact, accumulating evidence demonstrates that the increase of AGEs during the hyperglycemic condition leads to permanent abnormalities of endothelial cells, inducing the production of cytokines and reactive oxygen species through AGE-specific receptors [143,144]. These effects, as demonstrated in diabetic animal models and in humans, reflect the impaired vasodilatory response to NO [135], the reduced hyperemia [145], and the potentiation of platelet aggregation [146].

## 5. Conclusions and Recommendations

The association between diabetes and cardiovascular disease (CV) is now known. The micro- and macrovascular complications are in fact the main cause of morbidity and mortality in diabetic patients. In addition to dyslipidemia, obesity, and hypertension, which are considered the most common traditional CV risk factors in DM, hyperglycemia causes several alterations in endothelial and platelet function, which act as the real actors of diabetic vascular complications, not only contributing to the pathogenesis of vascular disease, but especially making difficult the clinical management of diabetic patients.

Unfortunately, until now, due to the complexity of diabetic pathology, most of the works have concentrated on how to intervene to minimize a single disease determinant, and only few studies have extended the experimental approaches evaluating the possibility to identify a single molecule able to control the two dysfunctional diabetic processes. In the light of existing data, we believe that study aimed at reducing both endothelial dysfunction and platelet hyperreactivity represents the best way to develop novel therapeutic strategies able to contain vascular diabetic complications, thus bringing both an increase of life expectancy and quality of life of diabetic patients.

## Abbreviations

DM	Diabetes Mellitus
CVD	Cardiovascular Disease
WHO	World Health Organization
eNOS	endothelial nitric oxide synthase
NO	nitric oxide
VCAM-1	vascular cell adhesion molecule
vWF	von Willebrand factor
CRP	C-reactive protein
TNF	tumor necrosis factor
Ang II	angiotensin II
PAI-1	plasminogen activator inhibitor-1
ADMA	asymmetric dimethyl arginine
LDL	low-density lipoprotein
HDL	high-density lipoprotein
VLDL	very low-density lipoprotein
NADPH	nicotinamide adenine dinucleotide phosphate oxidase
XO	xanthine oxidase
GTP	guanosine triphosphate
EC	endothelial cell
SREBP1	sterol regulatory element-binding protein 1
CD	cluster of differentiation
PKC	protein kinase C
HbA1c	glycated hemoglobin
AGEs	advanced glycation end products
RAGE	AGE receptor
GP	glycoprotein
P2Y12	purinergic signaling
cAMP	cyclic adenosine monophosphate
VASP-P	phosphorylation-dependent vasodilator-stimulated phosphoprotein
PK	protein kinase
IR	insulin receptor
IGF-1	insulin-like growth factor 1
Mac-1	macrophage-1 antigen
TF	tissue factor
COX	cyclooxygenase
TXA	thromboxane
HTPR	high on-treatment platelet reactivity
ADP	adenosine diphosphate
ACS	coronary syndrome
MI	myocardial infarction
PCI	percutaneous coronary intervention
DAT	dual antiplatelet therapy
PGI2	prostacyclin
Gi	G-protein inhibitory
